# Identification of Biochemical Network Modules Based on Shortest Retroactive Distances

**DOI:** 10.1371/journal.pcbi.1002262

**Published:** 2011-11-10

**Authors:** Gautham Vivek Sridharan, Soha Hassoun, Kyongbum Lee

**Affiliations:** 1 Department of Chemical and Biological Engineering, Tufts University, Medford, Massachusetts, United States of America; 2 Department of Computer Science, Tufts University, Medford, Massachusetts, United States of America; University of Virginia, United States of America

## Abstract

Modularity analysis offers a route to better understand the organization of cellular biochemical networks as well as to derive practically useful, simplified models of these complex systems. While there is general agreement regarding the qualitative properties of a biochemical module, there is no clear consensus on the quantitative criteria that may be used to systematically derive these modules. In this work, we investigate cyclical interactions as the defining characteristic of a biochemical module. We utilize a round trip distance metric, termed Shortest Retroactive Distance (ShReD), to characterize the retroactive connectivity between any two reactions in a biochemical network and to group together network components that mutually influence each other. We evaluate the metric on two types of networks that feature feedback interactions: (i) epidermal growth factor receptor (EGFR) signaling and (ii) liver metabolism supporting drug transformation. For both networks, the ShReD partitions found hierarchically arranged modules that confirm biological intuition. In addition, the partitions also revealed modules that are less intuitive. In particular, ShReD-based partition of the metabolic network identified a ‘redox’ module that couples reactions of glucose, pyruvate, lipid and drug metabolism through shared production and consumption of NADPH. Our results suggest that retroactive interactions arising from feedback loops and metabolic cycles significantly contribute to the modularity of biochemical networks. For metabolic networks, cofactors play an important role as allosteric effectors that mediate the retroactive interactions.

## Author Summary

Mathematical models are powerful tools to understand and predict the behavior of complex systems. However, the complexity presents many challenges in developing such models. In the case of a biological cell, a fully detailed and comprehensive model of a major function such as signaling and metabolism remains out of reach, due to the very large number of interdependent biochemical reactions that are required to carry out the function. In this regard, one practical approach is to develop simplified models that nevertheless preserve the essential features of the cell as a complex system by better understanding the chemical organization of the cell, or the layout of the biochemical network. In this work, we describe a computational method to systematically identify closely interacting groups of biochemical reactions by recognizing the modular hierarchy inherent in biochemical networks. We focus on cyclical interactions based on the rationale that reactions that mutually influence each other belong in the same group. We demonstrate our method on a signaling and metabolic network and show that the results confirm biological intuition as well as provide new insights into the coordination of biochemical pathways. Prospectively, our modularization method could be used to systematically derive simplified and practically useful models of complex biological networks.

## Introduction

Hierarchical modularity has emerged as an organizational principle of biochemical networks, where larger less cohesive clusters of network components (e.g. metabolic enzymes or signaling molecules) comprise functionally distinct sub-clusters [1], [2]. For example, Ihmels and coworkers analyzed the co-expression patterns of metabolic genes in *Saccharomyces cerevisiae* to find coordinated regulation of individual pathways as well as higher-order functions such as biosynthesis and stress response that require multiple feeder pathways [3]. Hierarchical organization was also observed by Gutteridge and coworkers for metabolic regulatory networks, where hub metabolites regulating many enzymes connect to modules of spoke metabolites that are chemically similar and/or regulate functionally related enzymes [4].

In recent years, observations on modularity have prompted metabolic engineers and synthetic biologists to consider whole pathways, rather than individual genes, as modular building units for cellular design [5]. An emerging design rule is to assemble and express a coherent set of genes that encode the desired biochemical pathway along with regulatory mechanisms that modulate the activity of the pathway [6]. Modularity analysis also offers a route to build practically useful, simplified models of complex biological systems. The size and complexity of biochemical networks reconstructed from genome databases has greatly increased over the years [7], [8], [9], rendering the estimation of kinetic or regulatory parameters either impractical or outright infeasible. In this regard, the modularity of a biochemical network should allow the system to be partitioned into minimally interdependent parts, enabling systematic derivation of coarse-grained, yet comprehensive models. Such coarse-grained models could greatly simplify the parameter estimation problem by substituting detailed reaction kinetics with less detailed module kinetics [10].

While there is general agreement that a biochemical module should represent a group of connected network components, and that the arrangement of modules in the network is hierarchical, there is less consensus on the criteria that should be used to systematically extract biologically meaningful modules [11], . One recent argument was to focus on cyclical, or ‘retroactive,’ interactions between network components, as opposed to simple connectivity [15]. Biochemical pathways operate with direction, where upstream components (e.g. concentration of reactants) influence downstream components (e.g. concentration of products). In the case where a downstream component also influences an upstream component (e.g. via a feedback regulatory mechanism), the two components participate in a cycle and thus interact retroactively. Placing such components into the same module reduces the interdependence between different modules, consistent with the intuitive definition of a biological module. Indeed, metabolic cycles and feedback loops have been shown to confer robustness [16] by isolating external perturbations and attenuating their propagation through the entire network [17].

**Figure 1:**
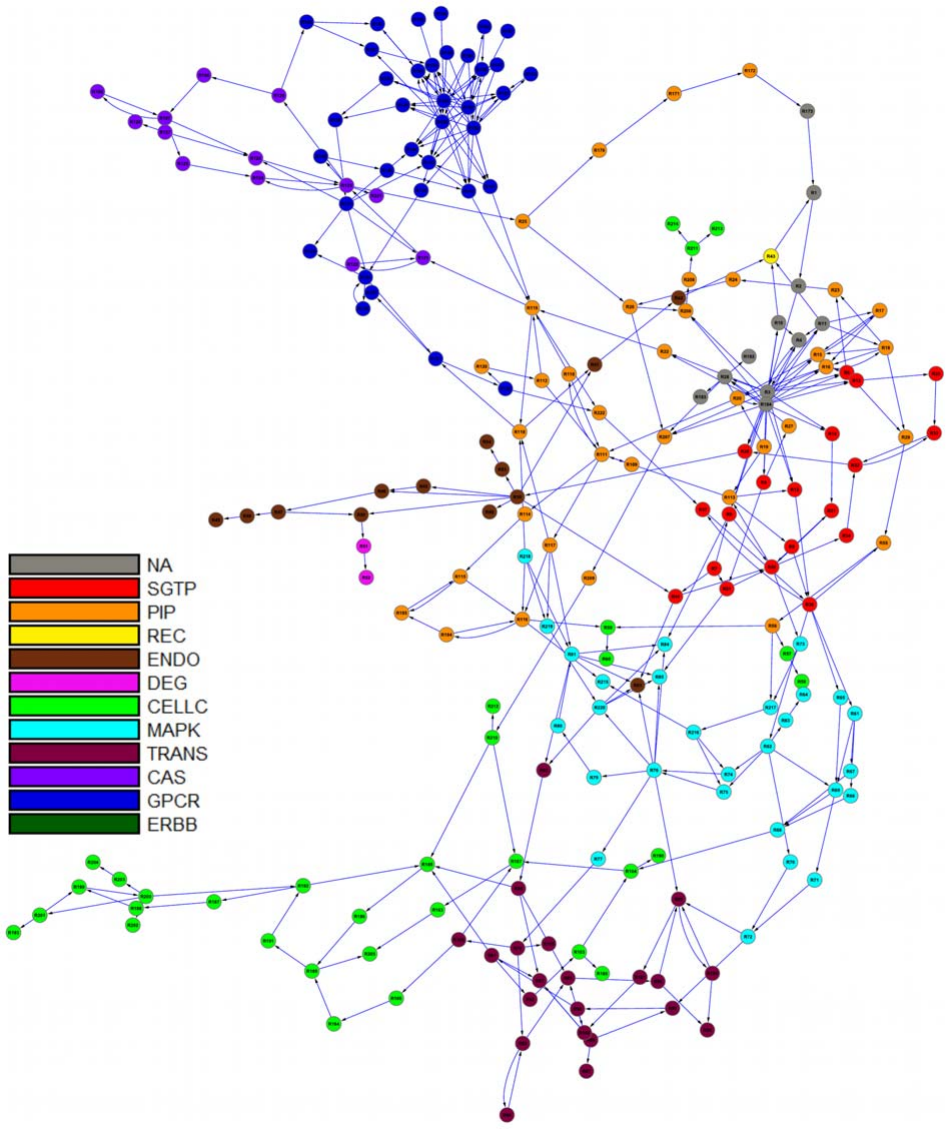
Graph image of the signaling network. Each reaction in the network was *a priori* assigned to one of 11 canonical signaling pathways as described in Methods. The pathway assignments are indicated by the color of the reaction vertex in the network. (NA: not assigned; SGTP: small guanosine triphosphatase mediated signaling; PIP: phosphatidylinositol polyphosphate signaling; REC: recycling; ENDO: endocytosis; DEG: degradation; CELLC: cell cycle; MAPK: mitogen-activated protein kinase cascade; TRANS: transcription; CAS: Ca^2+^ signaling; GPCR: G-protein coupled receptor mediated signaling; ERBB: erythroblastic leukemia viral oncogene homolog receptor signaling).

**Figure 2:**
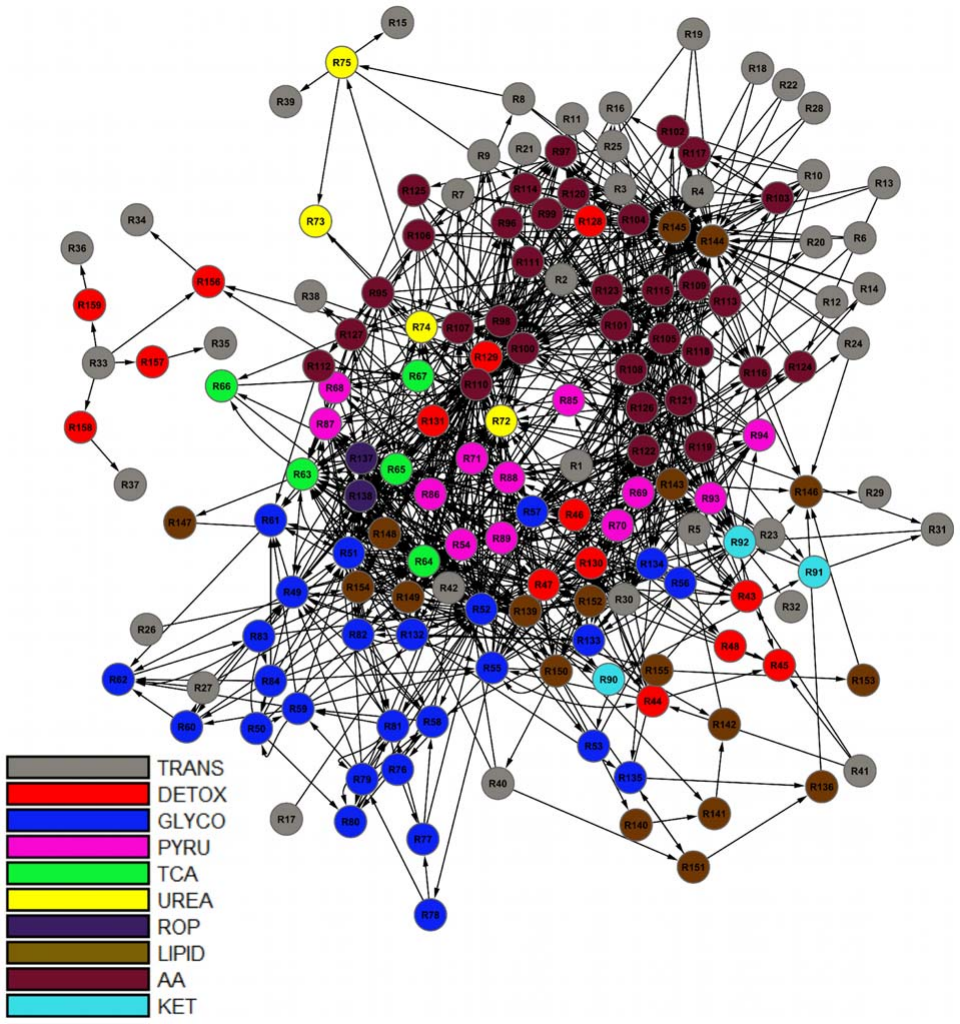
Graph image of the metabolic network. Each reaction in the network was *a priori* assigned to one of 10 textbook pathways as described in Methods. The pathway assignments are indicated by the color of the reaction vertex in the network. (TRANS: transport; DETOX: detoxification; GLYCO: glucose metabolism; PYRU: pyruvate metabolism; TCA: tca cycle; UREA: urea cycle; ROP: oxidative phosphorylation; LIPID: lipid metabolism; AA: amino acid metabolism; KET: ketone body metabolism).

In this paper, we extend the concept of retroactivity to account for cyclical interactions spanning distant parts of a biochemical network as exemplified by feedback loops of signaling and metabolic pathways. In earlier work [6], retroactivity was only considered for interactions between nearest neighbors in a network. To investigate hierarchy, we adopted Newman's algorithm for community detection [18] to successively partition a network into modules containing cyclical interactions based on a round trip distance metric, which we call Shortest Retroactive Distance (ShReD). Applied to test models of a signaling network [19] (Figure 1) and a metabolic network (Figure 2), the ShReD-based partitions produced hierarchically arranged modules that confirm biological knowledge. In addition, the partitions also revealed modules that are less intuitive. For the metabolic network, we also examined the role of allosteric regulators and cofactors as network elements that determine the number of cyclical interactions and the hierarchical depth of modules.

## Results

### Effect of Retroactive Interactions on Modularity: Signaling Network

**Figure 3:**
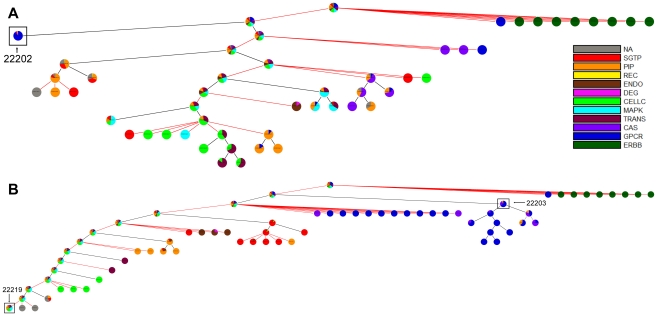
Hierarchical partitions of EGFR signaling network. (A) Partitions obtained using Newman's connectivity metric. The GPCR dominated module (ID: 22202) has 36 reactions and 28 cycles. (B) Partitions obtained using the ShReD metric. The GPCR dominated module (ID: 22203) has 39 reactions and 167 cycles. The terminal node (ID: 22219) has 99 reactions, but only 10 cycles.

**Figure 4:**
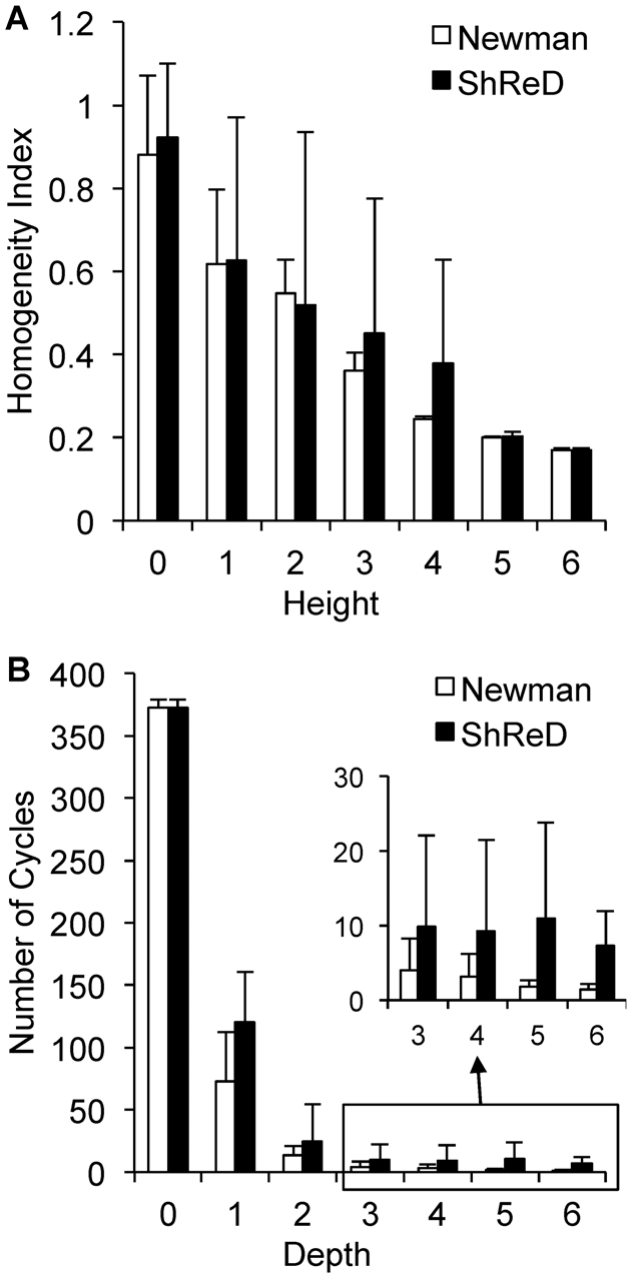
Effects of the partition metric on the properties of EGFR signaling network modules. (A) Homogeneity of modules as a function of partition height (see text in Methods for definition of homogeneity index). (B) Average number of cycles in a module as a function of network depth. Error bars represent one standard deviation.

To examine the effect of cyclical, i.e. retroactive, interactions on modularity, we compared the partitions of the EGFR signaling network obtained using Newman's connectivity (Figure 3a) and the ShReD metric (Figure 3b). Several qualitative similarities between the two partitions are evident. In both partitions, modules that possess a large fraction of reactions from phosphatidylinositol polyphosphate (PIP) signaling coupled to either intracellular Ca^2+^ signaling (CAS) or small guanosine triphosphatase (SGTP) were identified. Quantitatively, both partitions reach a hierarchical depth of 6 and become more homogeneous closer to the terminal nodes of the partition tree. From the root to terminal nodes, the canonical group compositions of the modules (represented by the pie colors) trend toward a single, dominant group (Figure 4a). At the terminal nodes (height zero), the fraction of reactions in a module belonging to a single canonical group, on average, exceeds 80% for both Newman and ShReD partitions.

There are also notable differences between the two partitions. While both partitions extract modules predominantly consisting of G-Protein coupled Receptor (GPCR) activation reactions, the ShReD partition identifies greater hierarchy stemming from those modules. In the Newman partition, there are several terminal leaf nodes that predominantly comprise Mitogen Activated Protein Kinase (MAPK) reactions. Analogous terminal nodes are not present in the ShReD partition. The ShReD partition yields a large terminal node consisting of 99 reactions (Supplementary Figure S1b, ID: 22219), whereas the largest terminal node of the Newman partition consists of 36 reactions (Figure S1a, ID: 22202). The largest terminal node in the Newman partition (ID: 22202) predominantly comprises GPCR transactivation reactions, whereas the largest terminal node in the ShReD partition (ID: 22219) comprises several signaling functions, including MAPK cascade, endocytosis, and cell cycle. Another notable difference is that while the average number of cycles in a module decreases with increasing depth for both partitions, a larger number of cycles are preserved in the ShReD partition at greater depths (Figure 4b).

### Effect of Retroactive Interactions on Modularity: Metabolic Network

**Figure 5:**
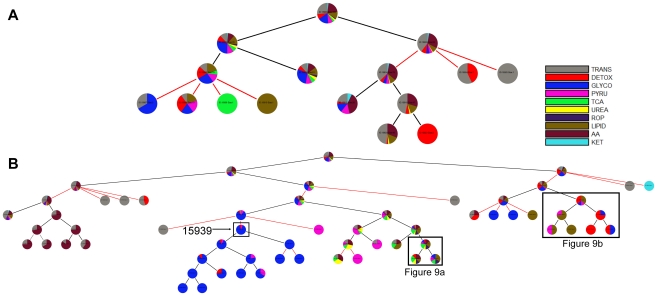
Hierarchical partitions of hepatocyte metabolic network. (A) Partitions obtained using Newman's connectivity metric. (B) Partitions obtained using the ShReD. Details of the reactions in the boxed modules are shown in Figures 9a and 9b. The other boxed module (ID: 15939) contains pyruvate kinase.

**Figure 6:**
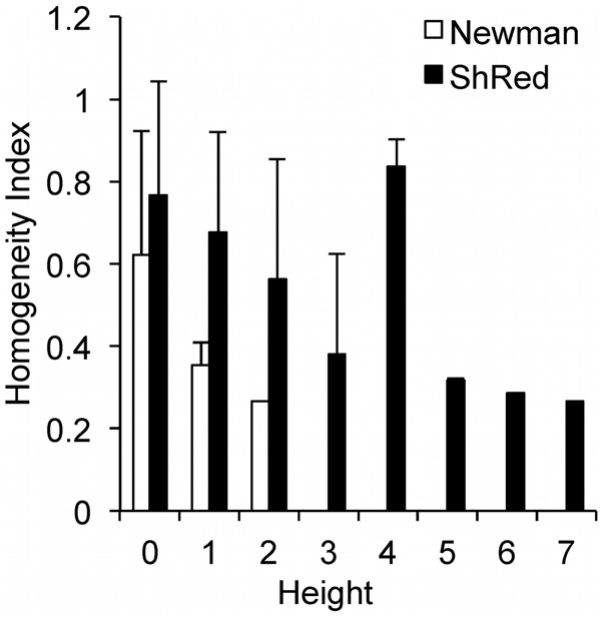
Effects of the partition metric on the properties of hepatocyte network modules. Homogeneity index is shown as a function of partition height. The height of the root node in the Newman partition tree is 2, whereas the height of the ShReD tree is 7. Error bars represent one standard deviation.

We next compared the Newman (Figure 5a) and ShReD partitions (Figure 5b) for the liver metabolic network, complete with regulatory edges and cofactors. As was the case for the EGFR network, both partitions lead to modules that generally increase in homogeneity from the root node to the terminal nodes (Figure 6). However, unlike the EGFR network, the arrangement and compositions of the two partitions are drastically different (Figure 5). In contrast to the Newman partition, the ShReD partition generates modules with hierarchical depth, similar to the GPCR dominated modules of the EGFR network. In the case of the metabolic network, hierarchical depth was greatest for modules comprising reactions in and around glycolysis (GLYCO). Moreover, the terminal node modules of the ShReD partition reach greater homogeneity compared to the Newman partition (Figure 6, Figure S2).

### Impact of Allosteric Regulation

**Figure 7:**
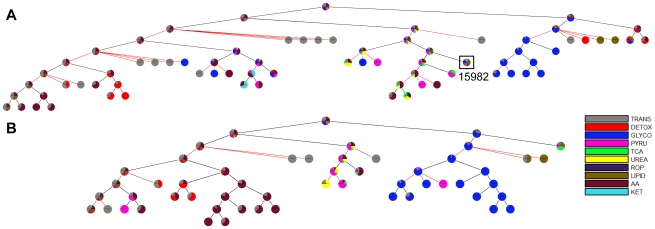
ShReD partitions of modified hepatocyte metabolic models. (A) Metabolic network with cofactors, but no regulatory edges. The boxed module (ID: 15982) contains pyruvate kinase. (B) Metabolic network with regulatory edges, but no cofactors. Note the absence of a redox module coupling detoxification reactions with lipid synthesis.

**Figure 8:**
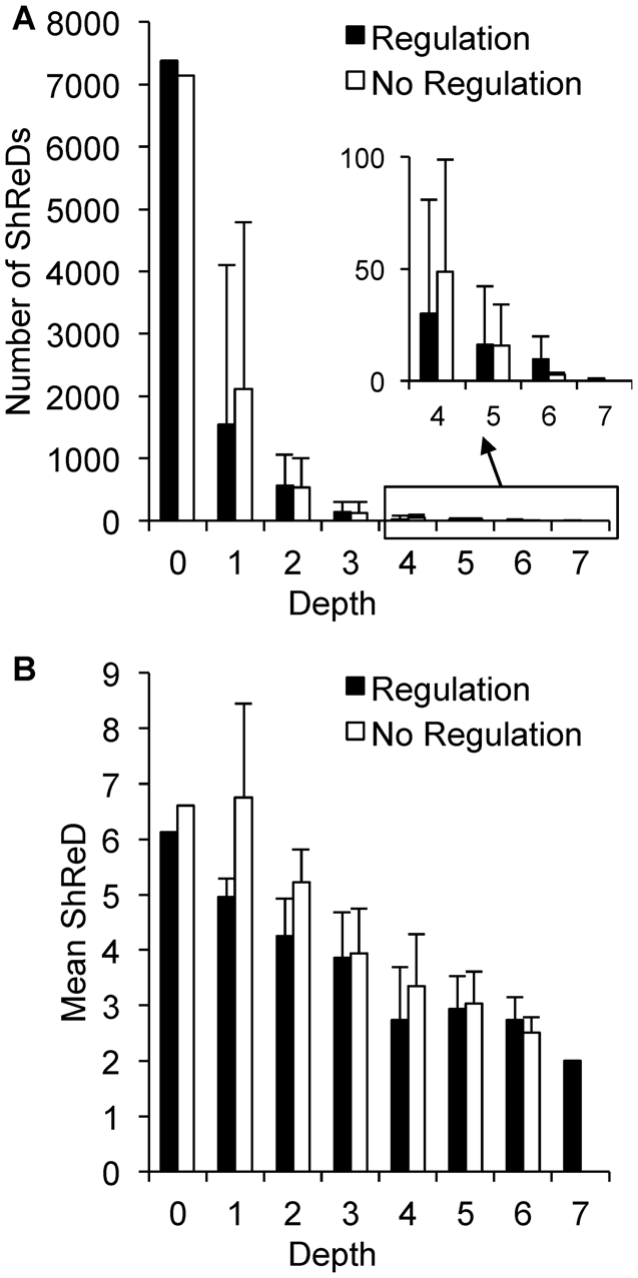
Effects of regulatory edges on module properties. (A) Number of finite ShReDs in a module as a function of partition depth. (B) Average ShReD of a module as a function of partition depth. Error bars represent one standard deviation.

The impact of metabolic regulation on ShReD-based modularity was investigated by comparing the partitions for the metabolic network model with (Figure 5b) and without the allosteric interactions (Figure 7a). The two models yield qualitatively similar hierarchical partitions with subtle differences in the placement of reactions into modules (Dataset S1). These differences include the placement of reactions coupled to the pyruvate kinase reaction, which is subject to a high degree of allosteric regulation relative to other reactions in the network. The quantitative impact of regulation is observed by comparing the number of ShReDs present in the network prior to the partition. At depth zero, there are approximately 250 additional ShReDs in the model with allosteric regulation compared to the model without regulation (Figure 8a). However, there is no obvious difference in the number of ShReDs between the two models at greater depths. There is also no obvious difference in the average ShReD at most depths, with the exception of depth zero, where the average ShReD is approximately 7% shorter for the model with allosteric regulation compared to the model without regulation (Figure 8b).

### Impact of Cofactors

**Figure 9:**
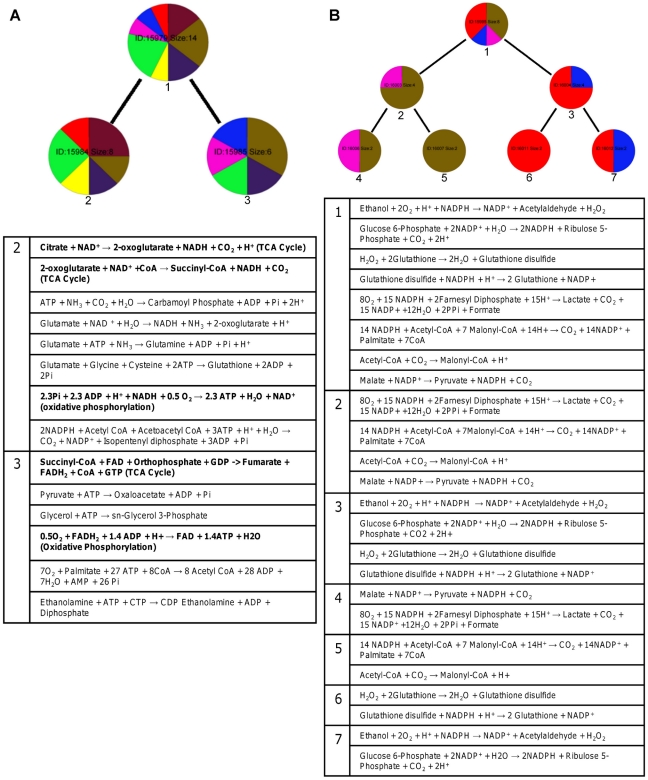
Redox modules. Detailed composition of modules boxed in Figure 5b. (A) Coupled reactions of the TCA cycle and oxidative phosphorylation (highlighted in bold type) metabolizing NADH and FADH_2_. (B) Coupled reactions metabolizing NADPH.

**Figure 10:**
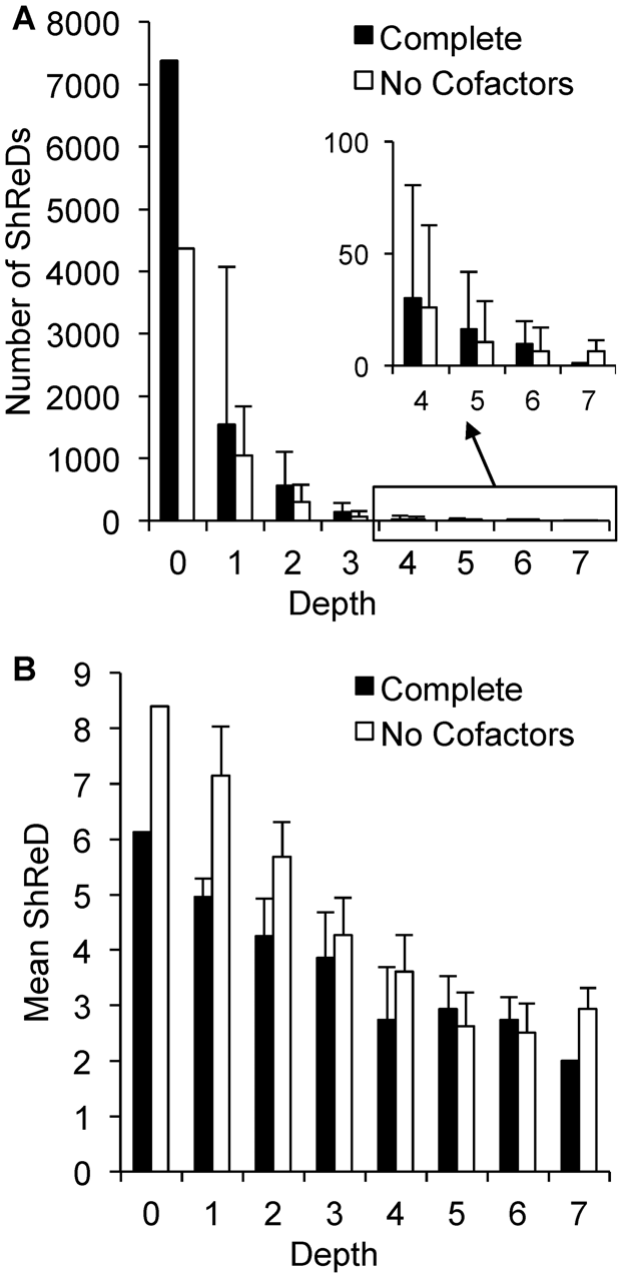
Effects of regulatory edges on module properties. (A) Number of finite ShReDs in a module as a function of partition depth. (B) Average ShReD of a module as a function of partition depth. Error bars represent one standard deviation.

We next assessed the impact of cofactors such as ATP, NADH, and NADPH on ShReD-based modularity by comparing the partition generated for the complete metabolic model (Figure 5b) to the partition for a partial model with regulatory edges, but lacking any interactions resulting from cofactors (Figure 7b). Qualitatively, the partitions reveal similar canonical groupings. Both partitions identify modules predominantly characterized by glucose metabolism (GLYCO) and modules predominantly characterized by amino acid metabolism (AA). Both partitions also group together reactions of the TCA cycle (TCA), urea cycle (UREA) and pyruvate metabolism (PYRU). A major difference between the two partitions involves the reactions of lipid metabolism (LIPID) and detoxification (DETOX). For the complete model, the ShReD partition identifies a module consisting of reactions from LIPID, DETOX, GLYCO, and PYRU (Figures 5b and 9b: ID: 15995), whereas no analogous module is identified for the model without cofactors. The reactions of module 15995 either produce or consume NADPH to support detoxification and lipid synthesis (Figure 9b). Quantitatively, the number of ShReDs trends lower when the cofactors are absent, with the largest difference observed at zero depth (Figure 10a). Conversely, the average ShReD of a module is generally larger when the cofactors are absent, with the largest difference again observed at zero depth (Figure 10b). At greater depths (>3), the average ShReD plateaus to a value between 2 and 3 edges for both models.

### Comparison with Local Retroactivity

For completeness sake, we compared the partitions based on ShReD with partitions based on local, or nearest neighbor, retroactivity. To obtain local retroactivity partitions, the size of cycles was restricted to two edges, effectively eliminating all retroactive paths involving non-neighboring vertices. Algorithmically, *ShReD_ij_* was set to infinity, if *ShReD_ij_* was greater than 2. Biochemically, a locally retroactive interaction represented either a reversible reaction catalyzed by a single enzyme or two irreversible reactions with opposite stoichiometry. For all cases, including the EGFR signaling network as well as various versions of the hepatocyte metabolic network, partitions based on local retroactivity failed to generate any modules.

## Discussion

In this paper, we introduce the use of ShReD as a round trip distance metric, which can be combined with a partition algorithm (adapted from Newman's earlier work on community detection) to systematically identify biochemical reaction modules that feature cyclical interactions. The notion of grouping together network components based on “retroactivity” was first proposed by Saez-Rodriguez and coworkers, who hypothesized that a strictly downstream component should have little impact on the activity of an upstream component unless there is a feedback or retroactive relationship [15]. It has been suggested that such feedback relationships contribute to robustness with respect to external perturbation, notably in signal transduction networks [16]. The ShReD metric accounts for cyclical interactions that span multiple reaction steps, and thus significantly extends on the prior work on retroactivity, which focused on local interactions between neighboring components. Previously, (shortest) path lengths between network components have been used to identify reaction modules by clustering, but without consideration of directionality and retroactivity [20].

To evaluate the performance of ShReD as a module-detection metric, we performed two sets of comparisons. One set of comparisons involved the community detection algorithm presented by Newman, which also formed the basis for our partitioning algorithm. Newman's original algorithm partitioned based on connectivity, and favored the placement of a pair of network elements (vertices in the graph representation) into the same module if the number of connections between the two elements exceeded the expected (e.g. average) number of connections assuming an equivalent network with edges placed at random. The second set of comparisons involved the special case of local feedback loops or cycles arising from reversible reactions. The results of these comparisons were used to investigate how multi-step signaling loops or metabolic cycles, as opposed to conventional connectivity or reaction reversibility, contribute to the modular organization of biochemical networks.

Applied to a model network of EGFR signaling, the ShReD-based partitions generated modules with a greater number of cyclical interactions across all depths compared to Newman's connectivity-based partitions (Figure 4b), consistent with the premise of the ShReD metric. Our results suggest that the total number of cyclical interactions in a network or module at least partially dictates the hierarchical depth of ShReD-based partitions. The ShReD-based partitions of the EGFR model generated one large terminal module with 99 reactions (Figure 3b, ID 22219), which could not be further modularized due to the relatively small number of cycles (a total of 10) in the module (∼0.5 ShReDs per reaction). In contrast, the GPCR dominated module (ID 22203) has 167 cycles and 774 ShReDs connecting just 39 reactions (∼20 ShReDs per reaction), and can be further partitioned to generate 4 additional levels of hierarchy.

In the case of the liver metabolic network, which has a substantially greater number of cycles (arising from allosteric feedback loops) compared to the EGFR signaling network, the difference between ShReD and Newman partitions is more dramatic. ShRed partitions again lead to greater hierarchy, reaching a depth of 7, whereas Newman's partition only reaches a depth of 3. The greater hierarchy achieved using the ShReD metric is significant, because the partition algorithm is essentially identical to Newman's algorithm, i.e. the only difference is the metric used to calculate the modularity score *Q*. For both metrics, a module is further partitioned only if the *Q* score is positive after the partition. Indeed, the scoring criterion based on the ShReD metric is actually more stringent, because the algorithm performs an additional test to ensure that the modules resulting from a partition each has at least one cycle. In this regard, the greater hierarchy generated by the ShReD (as opposed to the partition algorithm) gives credence to the metric for being able to identify hierarchical modules based on the preservation of cycles.

The retroactive interactions captured by ShReD include not only reaction reversibility (as in previous work [15]), but also cycles and feedback loops involving multiple reactions and allosteric effectors. Feedback loops resulting from allosteric regulation of an upstream enzyme by a downstream product represent an important regulatory motif that is common to biochemical networks. To examine the impact of feedback loops on modularity, ShReD partitions were obtained for the metabolic network with and without allosteric regulation. While qualitatively similar, the partitions differed in the placement of highly regulated reactions. For example, biochemistry textbooks generally associate pyruvate kinase (PK) with glycolysis, where the enzyme catalyzes the terminal step. The enzyme's activity is subject to allosteric regulation by several sugar phosphates produced upstream in glycolysis. On the other hand, the enzyme's product, pyruvate, is highly connected to the TCA cycle and amino acid pathways through anaplerosis and transamination reactions. When regulatory edges are absent, ShReD partitions place PK in one of the terminal leaf nodes along with reactions of lipid metabolism, pyruvate metabolism, the TCA cycle, oxidative phosphorylation and ketone body synthesis (Figure 7a, ID: 15982). When regulatory edges are present in the model, however, the partitions place PK in a module dominated by reactions of sugar metabolism (Figure 5b, ID 15939), consistent with textbook biochemistry. In this regard, the ShReD metric captures the impact of both stoichiometric connectivity and feedback regulation in determining modularity.

As many of the allosteric regulators were energy currency metabolites, we also examined the partitions for a partial metabolic model that lacks these cofactors. The resulting network contains fewer ShReDs, presumably reflecting an overall decrease in the total number of paths. Compared to the complete model, the corresponding ShReDs (connecting the same reaction vertices) of the partial model are ∼30% *longer*, indicating that allosteric feedback and other cofactor-dependent interactions more tightly couple the reactions in the network. In the present study, abstracting the metabolic network as a reaction-centric graph greatly facilitated the inclusion of cofactors in the modularity analysis, identifying both intuitive and non-canonical groupings that could not be identified by removing interactions effected by cofactors. For example, not including the cofactors in the model would completely isolate the oxidative phosphorylation reactions and carbamoyl phosphate production reaction from the rest of the metabolic network as disconnected components. Including the cofactors allows these reactions to be placed into modules; for the complete metabolic model, these reactions are kept together at a height of 2 (Dataset S1). Another example of cofactor-dependent modularity involves the association of NADH and FADH_2_ oxidation with different reactions in and around the TCA cycle (Figure 9a). The partitions place NADH oxidation into a module (ID: 15984) that also contains isocitrate and alpha-keto glutarate dehydrogenases, which are NADH producing reactions in the TCA cycle. Similarly, FADH_2_ oxidation is placed in a module (ID: 15985) containing succinate dehydrogenase, which reduces FAD^+^ to FADH_2_. The coupling between TCA cycle reactions and oxidative phosphorylation is intuitive. However, the TCA cycle reactions are also highly connected to reactions in glutamate metabolism and β-oxidation, associations that may be subjectively less intuitive. In this light, ShReD partitions reflect an emphasis on cyclical interactions mediated by the cofactors. A third example of an intuitive, yet non-canonical grouping involves the drug transformation reactions. In the present study, the metabolic model included reactions that are induced by troglitazone, a hydrophobic anti-diabetic compound withdrawn from the market due to severe hepaotoxicity. Module 15995 illustrates the cyclical interactions coordinating reactions of several different canonical pathways, including glutathione, lipid, glucose, and pyruvate metabolism (Figure 9b). A dominant characteristic (exhibited by seven of the nine reactions) of this module is the production and consumption of NADPH, again underscoring the significance of the cofactors in determining the modularity.

To examine whether the influence of the cofactors reflected the relatively small size of the model network (comprising ca. 150 reactions), we also applied the ShReD-based modularity analysis to a larger model of the human liver (comprising ca. 2500 reactions) [21]. The analysis again identified cofactor modules centered on NADH and NADPH consumption and production, similar to the smaller liver model (Figure S3, Dataset S2). Many of the terminal modules for the larger model comprised reactions that were grouped into analogous modules for the smaller model, suggesting that the size of the model did not qualitatively alter the structural organization of the metabolic network. Quantitatively, the maximum hierarchical depth was greater for the larger network, increasing from 7 to 16. The increased depth was presumably due to the greater detail of the HepatoNet1 model, which includes many additional pathways of amino acid, lipid and nucleotide metabolism.

In conclusion, this paper presents a novel methodology for modularity analysis that enables hierarchical partitions of biochemical networks by preserving feedback loops and other cyclical interactions. To the best of our knowledge, the present study is the first to build a module detection method that focuses on cycles or feedback loops as the key structural feature. The present study is also the first to account for cofactors in modularity analysis, further emphasizing the role of pathway regulation in network modularity. Previously, studies on modularity have generally ignored cofactors, citing methodological challenges arising from having to place these highly connected hub metabolites into particular modules [20], [22]. It should be noted that the current analysis, which does not weight the edges in calculating the ShReDs, implicitly assumes that all reactions in the network are equally engaged. Clearly, the levels of engagement can be expected to vary across different reactions, and should ideally be weighted appropriately, by using quantitative activity data such as metabolic flux. For example, a high glycolytic flux may confer a larger weight to edges representing PK regulation, which in turn may impact the overall modularity of the network. Moreover, cells subjected to different chemical or genetic perturbations will likely exhibit different flux dynamics, which would need to be reflected in the metric to obtain partitions that meaningfully analyze the modularity of a dynamic system such as the biological cell. A thorough examination of the role of reaction engagements in modularity analysis is beyond the scope of this study, and warrants further work in a future study.

## Methods

### Network Representation

**Figure 11:**
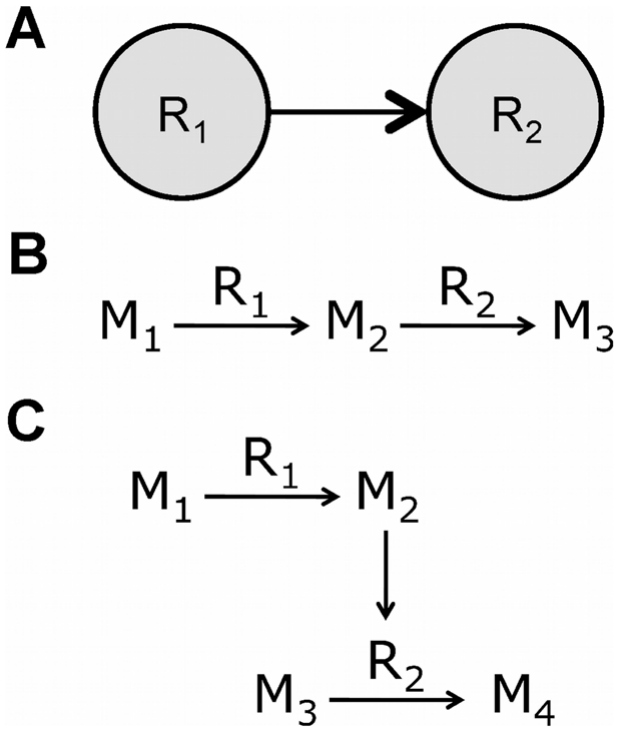
Network representation. (A) A reaction-centric representation of two different cases (B and C) where one reaction is upstream of another. (B) Reaction R_1_ produces a metabolite M_2_ that is consumed by reaction R_2_. (C) Reaction R_1_ produces a metabolite M_2_ that is an allosteric effector of the enzyme catalyzing reaction R_2_.

A common way to model a biochemical network using a graph is to represent the components as vertices and their interactions as edges. In this study, the focus is on understanding the hierarchical and modular relationship among reactions, treating metabolites as shared resources among modules. We therefore use a directed graph with vertices representing reactions and edges indicating a directional interaction between the connected reactions. Edges are drawn between two reactions (Figure 11a) if the product of one reaction is either a reactant (Figure 11b) or allosteric effector of another reaction (Figure 11c). For reversible reactions, reactant-product relationships are considered in both directions.

### Shortest Retroactive Distance

**Figure 12:**
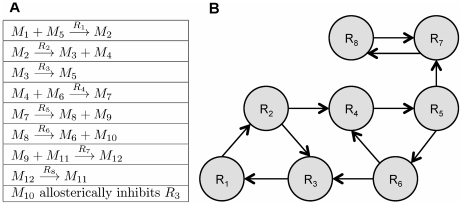
Example illustrating ShReD-based network partition. (A) The example network comprises 8 reactions and 1 allosteric inhibition. (B) Graph representation of the reaction-to-reaction interactions in the example network.

We utilize round trip distance as a metric, which we call Shortest Retroactive Distance (ShReD), to characterize the connectivity between two vertices that interact retroactively. A retroactive interaction exists between two vertices *i* and *j*, if and only if there is a directional path from vertex *i* to *j and* a return path from vertex *j* to *i*. The retroactive interaction represents a mechanism for mutual feedback, and thus expresses interdependence. The ShReD of vertices *i* and *j* (*ShReD_ij_*) is the sum of the *shortest path* distance from node *i* to *j* and the shortest return path distance from node *j* to *i*. In the example network of Figure 12, *ShReD_1,3_* is 3 because there are two edges along the shortest path from R_1_ to R_3_ and there is one edge from R_3_ to R_1_. There is another cycle connecting the two reaction vertices, which also involves R_4_, R_5_ and R_6_. This cycle, however, is not the ShReD, as its length of 6 exceeds the ShReD value of 3. For a given network (or sub-network) a ShReD value is computed for every pair of vertices in the network (or sub-network). To compute the ShReD values, we first calculated the shortest distances between all pairs of vertices using the Floyd-Warshall algorithm [23]. The resulting all-pairs shortest path matrix was then added to its own transpose to generate a symmetrical ShReD matrix. When there is no path or no return path between two vertices, the ShReD value between these two vertices is infinity. The ShReD between a node and itself is zero. For the example network in Figure 12, the ShReD matrix is as follows:
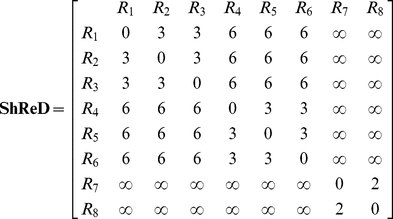
(1)


### Partitioning Algorithm

**Figure 13:**
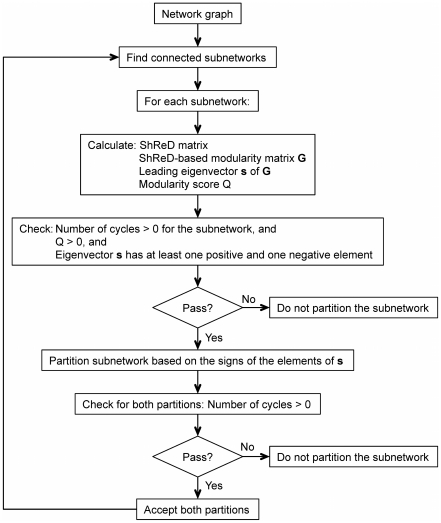
Schematic illustrating the flow of the partitioning algorithm.

Partitions were obtained by adapting Newman's community detection algorithm [18], which was modified to generate partitions based on the ShReD metric, as opposed to simple connectivity. An overview of the algorithm flow is shown in Figure 13. The initial step is to find the connected subnetworks in the parent network using a breadth-first traversal algorithm [24], as it is possible that the parent network, represented as a reaction centric graph, may not be connected. For the search, the network is represented as an undirected graph, as we are interested in identifying the connectivity of vertices, regardless of direction. Each connected subnetwork is then partitioned into two daughter subnetworks to maximize a “modularity score” while ensuring that each subnetwork resulting from a partition retains at least one retroactive interaction, i.e. cycle. Applied recursively, the algorithm produces a hierarchical tree of binary partitions.

In Newman's algorithm, the modularity score was computed as the difference between the actual and expected number of *connections* between two components. In this study, we computed the difference between the actual and expected ShReD to determine the modularity score. The expected ShReD between *i* and *j*, *P_ij_*, is computed as the arithmetic mean of the average of all non-zero and non-infinite ShReDs involving *i* and the average of all non-zero and non-infinite ShReDs involving *j*:
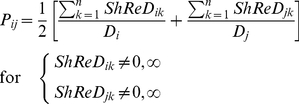
(2)where *D_i_* and *D_j_* are the number of non-zero and non-infinite ShReDs involving *i* and *j* respectively, and *n* is the total number of vertices in the network (or sub-network). We define a *ShReD-based modularity matrix*, **G**, as follows:
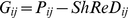
(3)The diagonal entries of **G** are set to zero, because both the expected and actual ShReD between a vertex and itself are zero. An entry *G_ij_* is also set to zero, if *ShReD_ij_* is infinity. For the example network in Figure 12, the average ShReD of R_1_ and R_2_ are both 4.8. The expected ShReD between R_1_ and R_2_, *P_12_*, is thus 4.8, and *G_12_* is 1.8. The full matrix **G** for the example network is shown below. The ShReD-based modularity matrix differs from Newman's *connectivity-based modularity matrix*, which does not take into account the direction of an interaction.
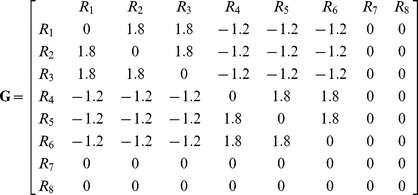
(4)Defining the modularity score *Q* based on the ShReD-based modularity matrix **G**, we wish to find a vector **s**, which assigns each vertex in the network to one of the two partitioned sub-networks to maximize *Q*:
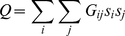
(5)where *s_i_* is an element of a vector **s**. Each *s_i_* has a value of either −1 or 1. An increase in *Q* is obtained in two cases: if *G_ij_* is positive and the vertices *i* and *j* are assigned to the same sub-network (*s_i_* = *s_j_* = 1 or *s_i_* = *s_j_* = −1), or if *G_ij_* is negative and the two vertices are assigned to different subnetworks (*s_i_* = 1 and *s_j_* = −1 or vice versa). The vector **s** maximizing *Q* can be found using spectral partitioning methods [25] as described by Newman [18]. The solution to the maximization problem can be approximated by the leading eigenvector of **G**. For our example network (Figure 12), the leading eigenvector of **G** (Equation 4) is given by **v** = [−0.41, −0.41, −0.41, 0.41, 0.41, 0.41, 0, 0], from which **s** is approximated as **s** = [−1, −1, −1, 1, 1,1, −1, −1]. All non-positive entries, including zero, in the eigenvector are assigned the value −1. This partition assigns R_1_, R_2_, R_3_, R_7_ and R_8_ to one module, and R_4_, R_5_ and R_6_ to the other module. The reactions in the first module are not fully connected, which gives rise to two disconnected components, one comprising R_1_, R_2_ and R_3_ and the other comprising R_7_ and R_8_. In this example, a single binary partition generated three separate modules, each consisting of a single cycle.

In Newman's original community detection algorithm, partitioning of a subnetwork continues if the modularity score *Q* is greater than zero and the leading eigenvector **s** of the modularity matrix **G** has at least one positive and one negative element; otherwise the subnetwork is not further partitioned. The algorithm terminates if there is no subnetwork that can be further partitioned. In our algorithm, we modified the termination criterion to also check that there is a cycle in each subnetwork resulting from a partition operation. The check for a cycle was performed using an algorithm similar to topological sort [26]. For a given module abstracted as a directed graph, the number of incoming edges is computed for each vertex. A vertex with zero incoming edges is removed from the graph along with its outgoing edges. The number of incoming edges is then recalculated for the remaining vertices. The process repeats until there are no more vertices, in which case the graph has no cycles, or until there are no vertices with zero incoming edges, indicating the presence of a cycle. In our example, the *Q* score for the first partition is greater than zero (*Q* = 43.2) and each resulting subnetwork contained at least one cycle. Thus, the partitioned subnetworks are accepted as modules and the algorithm continues by finding the connected subnetworks in each module. The module comprising R_1_, R_2_, R_3_, R_7_ and R_8_ is not fully connected, and two subnetworks are found, one comprising R_1_, R_2_, and R_3_ and the other comprising R_7_ and R_8_. Neither subnetwork can be further partitioned, as every element in the leading eigenvector of the corresponding modularity matrix has the same sign. Similarly, the module comprising R_4_, R_5_ and R_6_ cannot be further partitioned, as every element in the leading eigenvector of the corresponding modularity matrix has the same sign, and the algorithm terminates.

### Hierarchical Tree of Partitions

The partitioning results are reported in the form of a hierarchical tree annotated with several properties. Each module is represented as a pie chart, where the size of each slice is proportional to the fraction of reactions that belong to the corresponding, pre-assigned canonical (textbook) grouping. The *homogeneity index* of a module corresponds to the fraction occupied by the largest slice in the pie chart. The homogeneity index therefore ranges from 0 to 1, where a larger number indicates greater homogeneity in terms of composition based on the canonical group assignments. The black lines connecting the nodes in the hierarchical tree represent ShReD-based partitions, whereas the red lines represent the formation of components from partitions that include disconnected components. The depth of a module is determined as the number of black edges traversed from the root node to the module. The height of a module is determined as the largest possible number of black edges traversed from the module to a terminal leaf node.

The *number of cycles* within a module is used to compare the partitions obtained based on the ShReD and Newman's connectivity metrics. While standard algorithms exist for counting the number of cycles in a graph [27], the run time is proportional to the number of (non-unique) cycles. The number of cycles may be exponential in the number of vertices, and renders cycle counting as computationally inefficient. The cycle count is thus reported up to 1,000 unique cycles. Any count above 1,000 is effectively reported as 1,000. In addition to cycles, we also determined the number of *non-infinite* shortest retroactive paths in a module as well as the mean ShReD of the module. The mean ShReD of a module is calculated by averaging the corresponding non-infinite entries in the corresponding ShReD matrix.

### Models

As case studies, we examined two types of biochemical networks that feature directed interactions and feedback loops.

#### Signaling model

The signaling network was reconstructed based on a published model of epidermal growth factor receptor (EGFR) signaling [19]. The model was downloaded as an SBML file and cast into the form of a stoichiometric matrix based on the directional interactions between signaling molecules defined in the SBML file. The model consisted of 322 signaling molecules (metabolites and proteins) participating in 211 signaling reactions. In addition to the signaling reactions, the model includes 238 allosteric interactions between the signaling molecules and reactions. The reactions in this model were *a priori* assigned to groups based on their previously catalogued function [19]. For example, the reactions that convert ERK1/2 and PKB/akt into their active forms were assigned to the MAPK cascade and PIP signaling, respectively. This initial grouping, which reflects historical knowledge of signaling modularity, provided a basis for comparison between biological knowledge-driven, canonical associations versus partition-driven, systematically obtained network modules.

#### Metabolic model

A stoichiometric network model of human hepatocyte metabolism was reconstructed from the KEGG reaction database and further augmented by the addition of xenobiotic transformation reactions, as well as regulatory interactions mediated by allosteric effectors. The model comprised 159 reactions, 146 metabolites, and 61 regulatory interactions. The xenobiotic transformation reactions were added to describe the metabolism of the anti-diabetic compound troglitazone (TGZ), including steps needed to supply conjugation substrates such as glutathione (GSH). The regulatory interactions in the model reflect known allosteric effects of metabolites on reaction activity as described in a standard biochemistry textbook [28]. Information about the allosteric effects of metabolites was organized into a regulatory matrix, where the columns and rows represented the effector metabolites and reactions regulated by these metabolites, respectively. The inhibition or activation of a reaction *j* by an allosteric effector *i* was denoted by a negative one (−1) or positive one (+1), respectively, in the matrix element (*i*, *j*). For all other cases where there were no known allosteric interactions, a zero (0) was entered into the corresponding matrix element. For example, the TCA cycle intermediate citrate allosterically inhibits phosphofructokinase I (PFK) to regulate the flux of glucose-derived substrates into the TCA cycle. In the regulatory matrix, this feedback inhibition is denoted by the value (−1) in the matrix element corresponding to (*citrate*, *PFK*). Similar to the EGFR signaling model, the reactions of the metabolic model were assigned into one of the following groups based on their canonical memberships as defined in standard biochemistry textbooks: transport (TRANS), detoxification (DETOX), sugar metabolism (encompassing glycolysis, gluconeogenesis, pentose phosphate shunt, and glycogen metabolism) (GLYCO), pyruvate metabolism (PYRU), TCA cycle (TCA), urea cycle (UREA), oxidative phosphorylation (ROP), lipid metabolism (LIPID), amino acid synthesis and degradation (AA), and ketone body production (KET). To test the impact of allosteric regulation on modularity, separate graph models were constructed by either omitting regulatory edges altogether, or just removing the cofactors (i.e. ATP, NADH and NADPH), which represented the majority of allosteric effectors. The cofactors were removed from the stoichiometric network model by deleting the corresponding rows of the stoichiometric matrix, which eliminated the regulatory edges reflecting cofactor-driven dependencies between reactions.

#### HepatoNet1

To investigate the effect of scale, a more detailed graph model of human liver metabolism was constructed from a previously published model (HepatoNet1) [21]. The HepatoNet1 model was downloaded as an SBML file and cast into the form of a stoichiometric matrix based on the reaction definitions in the SBML file. The model comprised 2539 reactions and 777 metabolites. This model was used as is, without further addition of allosteric regulatory interactions.

## Supporting Information

Dataset S1 Partition report for the hepatocyte metabolic model. The report includes the reaction definitions, regulatory interactions, and stoichiometric matrix for the model.
(XLSX)

Dataset S2 Partition report for the Hepatonet1 model. The report includes the reaction definitions and stoichiometric matrix for the model.
(XLSX)

Figure S1 Network of terminal modules from the partitioning of the EGFR signaling network using Newman's connectivity (A) and ShReD (B). The interactions between modules represent interactions between reactions in the respective modules. The size of a module is proportional to the number of reactions in the module. As the networks correspond to the terminal nodes of the respective partitioning trees, hierarchical information can be inferred from the presence of multiple modules assigned to the same canonical signaling pathway. For example, panel B shows multiple GPCR transactivation modules (dark blue) of varying sizes. In the same panel, MAPK cascade (light blue) is present as a component of a larger composite module with multiple canonical signaling pathways.
(TIF)

Figure S2 Network of terminal modules from the partitioning of the hepatocyte metabolic network based on both Newman's connectivity metric (A) and ShReD (B). The interactions between modules represent interactions between reactions in the respective modules. The size of a module is proportional to the number of reactions in the module.
(TIF)

Figure S3 ShReD based partitioning of Hepatonet1 model. Boxes highlight modules centered on NADH (module ID: 253956) and NADPH (module ID: 254789) consumption and production. The two modules share a number of reactions with identical main (carbon) reactants but different cofactors. For example, malate oxidation in the mitochondria (r0057) is in the NADH module, whereas malate oxidation in the cytosol (r0058) is in the NADPH module.
(TIF)
