# Supplementary figures and images for: Identification of Biochemical Network Modules Based on Shortest Retroactive Distances
Source: PLoS Comput Biol. 2011 Nov 10;7(11):e1002262. doi: 10.1371/journal.pcbi.1002262 (PMC3213171; doi:10.1371/journal.pcbi.1002262)

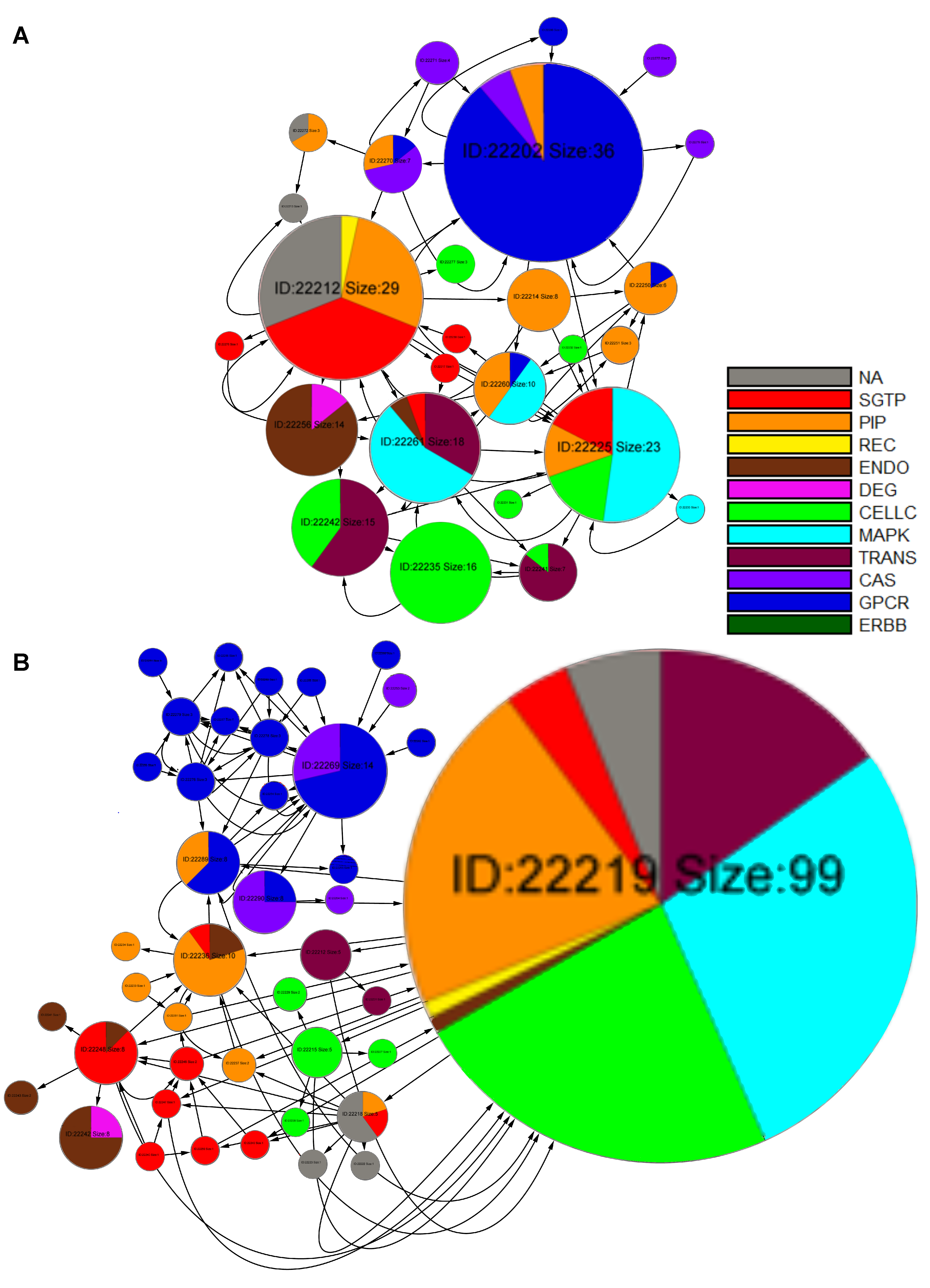

Supplement: Figure S1 — Network of terminal modules from the partitioning of the EGFR signaling network using Newman's connectivity (A) and ShReD (B). The interactions between modules represent interactions between reactions in the respective modules. The size of a module is proportional to the number of reactions in the module. As the networks correspond to the terminal nodes of the respective partitioning trees, hierarchical information can be inferred from the presence of multiple modules assigned to the same canonical signaling pathway. For example, panel B shows multiple GPCR transactivation modules (dark blue) of varying sizes. In the same panel, MAPK cascade (light blue) is present as a component of a larger composite module with multiple canonical signaling pathways. (TIF) [file pcbi.1002262.s003.tif]

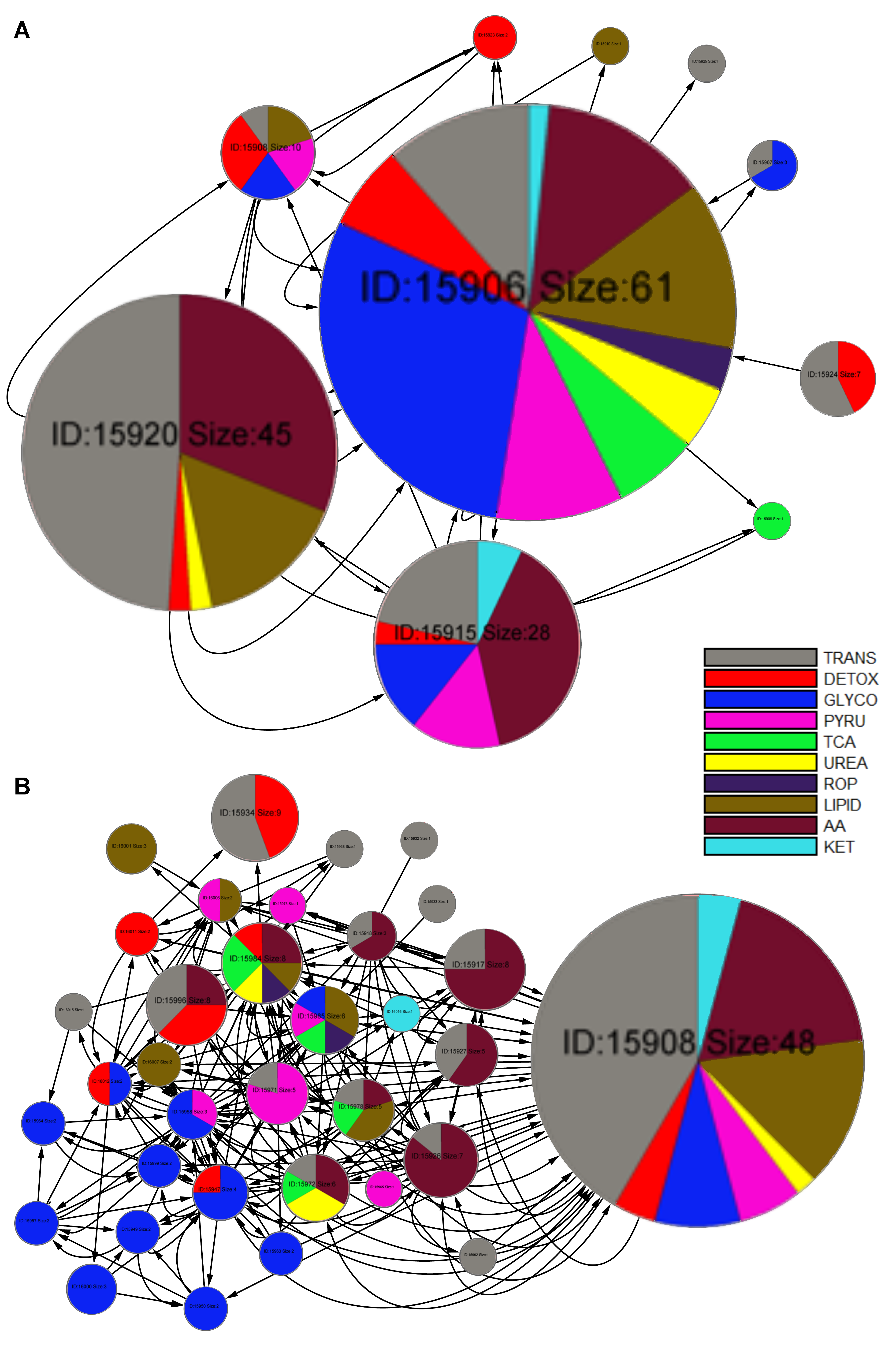

Supplement: Figure S2 — Network of terminal modules from the partitioning of the hepatocyte metabolic network based on both Newman's connectivity metric (A) and ShReD (B). The interactions between modules represent interactions between reactions in the respective modules. The size of a module is proportional to the number of reactions in the module. (TIF) [file pcbi.1002262.s004.tif]

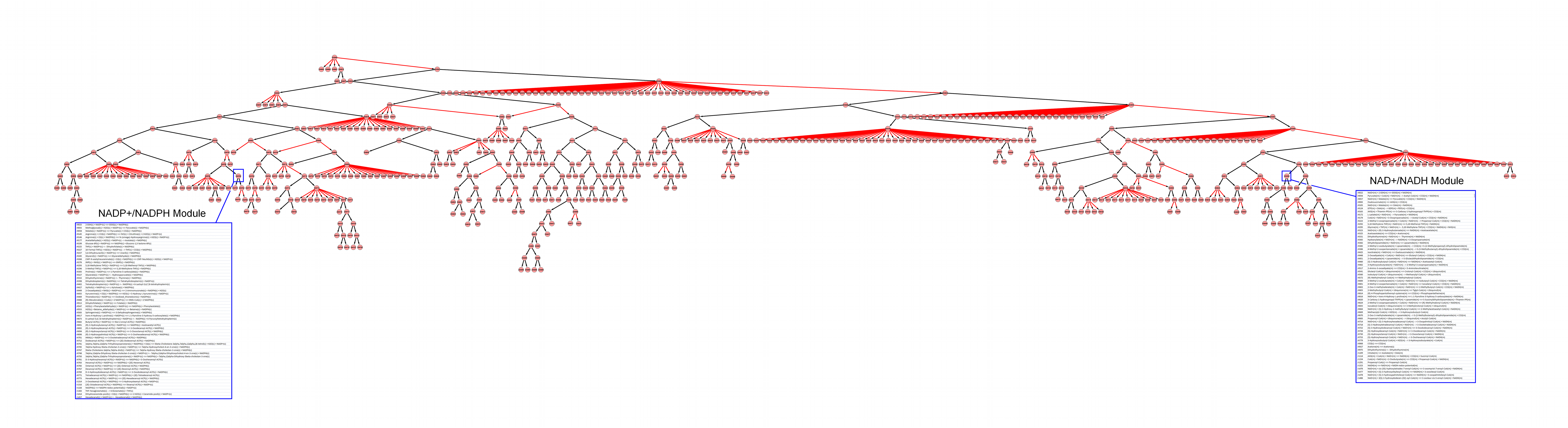

Supplement: Figure S3 — ShReD based partitioning of Hepatonet1 model. Boxes highlight modules centered on NADH (module ID: 253956) and NADPH (module ID: 254789) consumption and production. The two modules share a number of reactions with identical main (carbon) reactants but different cofactors. For example, malate oxidation in the mitochondria (r0057) is in the NADH module, whereas malate oxidation in the cytosol (r0058) is in the NADPH module. (TIF) [file pcbi.1002262.s005.tif]
